# Experiences with teachers in childhood and their association with wellbeing in adulthood

**DOI:** 10.1186/s40359-022-01000-6

**Published:** 2022-12-01

**Authors:** Christian Dittmann, Simon Forstmeier

**Affiliations:** 1 Developmental Science and Special Education, Department of Educational Sciences, Faculty II, University of Siegen, Siegen, Germany; 2 Developmental Psychology and Clinical Psychology of the Lifespan, Department of Psychology, University of Siegen, Adolf-Reichwein-Str. 2a, 57068 Siegen, Germany

**Keywords:** Experiences with teachers, Well-being, Self-esteem, Teacher-student relationship, Meaningful experiences

## Abstract

**Background:**

Prior research mainly focussed on the impact of the teacher-student relationship on teachers emotions and wellbeing. Current data shows a relationship between the quality of the teacher-student relationship and children's mental health. Unfortunately, it has not yet been investigated whether meaningful experiences with teachers also have an impact on students' well-being and whether an effect can still be found in adulthood. This work examines the impact of meaningful experiences with teachers during childhood and adolescence on the well-being of adults.

**Methods:**

The data in this study was collected by using a questionnaire. The current well-being of the participants was assessed with measures of life satisfaction, resilience, anxiety, stress, depressiveness, and self-esteem. Also, participants were asked to briefly write about their most meaningful experiences with teachers and rate them regarding their valence. These experiences were categorized into seven categories using a summarizing content analysis. We then conducted a statistical analysis with the data obtained.

**Results:**

The results showed a highly significant correlation between the participants’ self-esteem and the valence ratings of their experiences. Furthermore, the experience category had a substantial effect on individual self-esteem. Overall, this study demonstrated that a relationship exists between the well-being of adults and their experiences with teachers during childhood and adolescence.

**Conclusion:**

The results of this study call for a reflective, fair, authentic, and empathetic approach to students. Accordingly, teachers should be intensively trained to establish a relationship with their students that is characterized by appreciation and empathy.

## Experiences with teachers in childhood and their association with wellbeing in adulthood

Prior studies on teacher-student relationships and the emotions involved were mainly focused on the teachers’ emotions [1–5]. These studies showed that the students’ emotions have an impact on the emotions of the teachers. Furthermore, they showed that students’ emotions are associated with their learning outcomes [6]. However, the present study builds on prior research with a focus on students' emotions within the context of their interaction with teachers, which has received little attention to date.

Numerous studies performed investigated the effects of wellbeing. Various positive outcomes of a high rate of wellbeing have been measured, such as mental and physical health benefits. Diener and Chan [7] performed a systematic review on this topic and pointed out, that higher wellbeing predicts less cardiovascular diseases, a lower blood pressure, lower pain and a higher tolerance to pain. Furthermore, participants reported a better life quality and showed a faster wound healing. These positive effects of wellbeing even lead to less mortality. This emphasizes the importance of wellbeing in achieving the goal of living a long and healthy life.

### Teacher-student relationship and mental health

Resnick et al. [8] investigated the connection between teacher-student relationships and the mental health of the students. They showed that a significant association exists between a positive and supporting teacher-student relationship and students’ mental health. Those students with a supportive bonding to a teacher were less likely to complete suicide, had fewer suicidal thoughts, and had a lower level of stress. Furthermore, these students committed fewer crimes and were less frequently addicted to drugs than students having negative relationships with teachers.

In a Norwegian study conducted by the World Health Organization in 2003, a correlation between the general life satisfaction of students and the teacher-student relationship was shown. A total of 887 students were interviewed in the study, as revealed, students who received special support from a teacher were significantly happier. The study also found that the stress level of these students was significantly lower [9].

The previous studies show that students’ relationship with the teacher has a measurable influence on life satisfaction, stress perception, and emotions in general. Students who receive special support from the teacher are happier, less stressed, and commit suicide much less frequently. The relationship with the teacher acts as a predictor of mental health and is related to the frequency of drug abuse and outbreaks of violence.

It remains questionable whether these results are stable and whether the teacher-student relationship has an effect in adolescence and young adulthood. To this end, Wang et al. [10] conducted a study in 2013. They investigated whether the teacher-student relationship is related to depression in adolescence. A total of 1400 adolescents were examined in the survey. It was found that those adolescents who had a positive relationship with a teacher were significantly less likely to suffer from depression. This effect could also be measured in the 18th year of life.

### Teacher-student relationship and stress

Considering previous researches on stress level resulting from the teacher-student relationship, it is noticeable that they focused more on the teacher's stress level [11–16]. Nevertheless, some of these findings are also of interest to this study. For example, it was shown that the stress level of the teacher has a direct influence on the relationship with students. If teachers had a high stress level, the relationship with their students tends to be negative [16]. It remains questionable to what extent the stress level of the student is influenced by the interaction with their teacher.

Another study investigated the extent to which the stress levels of first-grade primary school students are influenced by their teachers’ relationship with them. Saliva samples were collected from 105 (n = 105) students on Monday and Friday and examined for cortisol levels. In this way, the researchers were able to measure the stress levels during the week. In order to assess the teacher-students relationship, teachers were asked to document various aspects such as closeness, conflicts, etc., and closely observe their students over the course of the week. Comparing the stress level of students in a supportive relationship with the teacher and the stress level of students in a conflictual relationship shows that a positive relationship leads to lower cortisol levels. It is interesting to note that students in a balanced relationship; between conflict and support had the lowest cortisol levels [17]. It remains questionable to what extent this influence can be measured over a longer period.

### Teacher-student relationship and self-esteem

A study conducted in Ghana measured the extent to which the university student–lecturer relationship influences students’ self-esteem. The researchers used two measuring instruments: the Rosenberg Self-Esteem Scale (SES) and the Student Instructor-Relationship Scale (SIRS). Interestingly, although the measurements showed a correlation between a positive lecturer-student relationship and higher self-esteem, it is not statistically significant. However, it was revealed that a positive relationship correlated with better academic performance, which in turn correlated significantly with higher self-esteem [18]. Although the results are not significant in terms of self-esteem, there is a tendency for the teacher-student relationship to affect self-esteem. This tendency was confirmed from an earlier study in 1994, in which a total of n = 606 young adults were asked to describe their relationship with teachers, parents, and friends. At the same time, self-esteem and school success were measured. Interestingly, the researchers concluded that relationships with parents, friends, and teachers significantly influence self-esteem [19].

The state of the research suggests that the teacher-student relationship contributes to the development of a healthy self-esteem. However, even in the studies reviewed here, only the immediate effect was examined. Here too, it remains questionable whether the relationship has a long-term influence on self-esteem or the effect fades away after a short time.

### Teacher-student relationship and students’ resilience

Several studies indicate that positive social relationships correlate with an individual’s resilience. It has been shown that at least one positive relationship is necessary to increase mental resilience [20]. Current studies on the effect of the relationship on the resilience of students are very few. Johnson [21] used qualitative data from an Australian longitudinal study conducted from 1997 to 2005. In this study, interviews were conducted with different students and then repeated four years later. The interviews were conducted as guided interviews and dealt with various topics that helped to assess resilience. For example, students were asked about important events in their lives, who is most important to them, and whether they achieved their goals or their plans were implemented. They were also asked what may have prevented them from achieving certain goals. Overall, this research showed that students with positive teaching experience had a higher degree of resilience. Of particular interest are the characteristics of a teacher that promotes resilience. Such teachers are particularly accessible to their students, cared about their students’ worries and fears, and listen actively to them. Mostly mentioned is that one teacher was particularly supportive, especially in the subject lessons. Students who had problems with reading or writing, for example, benefited from a teacher who took special care of these students and supported them in improving these basic skills. This special support had a positive effect on their ability to learn independently and acquire more skills. Furthermore, a helpful teacher-student relationship was characterized by a high degree of empathy. Teachers with the ability to understand and empathize were particularly helpful in developing resilience. Referral to professionals on specific issues was also cited as an important resource. It was also emphasised that teachers should prevent bullying and exclusion in schools [21].

The characteristics of a teacher that helps to promote resilience as described in these qualitative studies are strongly reminiscent of the basic attitudes described by Carl Rogers [22]. The variables of empathy, acceptance, and congruence are still considered to be fundamental therapeutic attitudes today. Empirical findings on the connection between positive experiences relationship and pronounced resilience, as well as the qualitative results regarding the teacher-student relationship, indicate that the promotion of resilience is also a key factor in the success of teacher-student relationships. However, it remains questionable whether the positive and negative effects of the teacher-student relationship are stable over time or how the quality of the teacher-student relationship and the experiences with teachers during childhood and adolescence affect the psychological state of adults.

### Aim of the present study

The aim of our study was to find out which experiences students made with their teachers during their childhood and adolescence and if these still affect their well-being today.

Considering the results presented above, we hypothesized that there is a connection between meaningful experiences with teachers in childhood and adolescence and the well-being of adults, i.e., their resilience, self-esteem, life satisfaction, and level of stress, anxiety, and depression.

#### Hypothesis 1

Adults with mostly positive experiences with teachers in their childhood and adolescence have higher ratings of resilience, self-esteem, and life satisfaction than those with mostly negative experiences.

#### Hypothesis 2

Adults with mostly negative experiences with teachers will have higher ratings of stress, anxiety, and depression.

#### Hypothesis 3

There will be a difference in these variables according to the categorization of the reported experiences. For instance, adults with experiences of violence will have a higher level of stress, anxiety, and, depression and lower resilience, life satisfaction and self-esteem than those who report experiences categorized as empathic, supporting, or motivating.

## Methods

### Participants

The study participants are at least 18 years old. This was a necessary limitation, as the longer-term impact of the teacher-student relationship on the well-being of adults was investigated. In addition, the participants had to have good German language skills and have gone through the German school system.

Study participants were recruited via mailing lists and notes on the notice boards at the XXX, from different groups in social media networks like Facebook and Instagram. Through a call on the website "Survey Circle," more individuals were informed about the study.

As an incentive for participation for all participants, three coupons of ten euros each for an online shop were drawn in lots. Therefore, the study participants were motivated to leave their e-mail addresses at the end of the survey.

#### Informed consent

The objectives and goals, detailed information about assessment and intervention, and the procedure of randomisation were explained to the participants. Written informed consent was obtained from all participants prior to their inclusion. The study protocol was approved by the ethics committee of the University of Siegen.

### Materials

Current well-being was assessed using measures of life satisfaction, stress, anxiety, depression, self-esteem, and resilience.

#### Life satisfaction

The Satisfaction with Life Scale (SWLS), developed by Diener et al. [23], was used to assess life satisfaction of the participants. The SWLS consists of five items and has evolved into a frequently used measurement tool in psychological research because of its high reliability and internal validity. Cronbach’s alpha is 0.86 in the present sample. In addition, the results of this questionnaire tend to correlate highly with the results of other measures with similar survey objectives and can be applied to different age groups [23].

#### Self-esteem

The Rosenberg Self-Esteem Scale (SES) developed by Morris Rosenberg [24] was used to measure participants current self-esteem. Initially developed to measure only the self-esteem of high school students but is now used to measure all age groups. It is characterized by a high reliability and validity and produces results that correlate strongly with similar measuring instruments [24]. The internal consistency of the SES in this sample was high with a Cronbach’s α of 0.90. Studies have also established a correlation between the results of the SES and the results of questionnaires used to survey anxiety and depression [25].

#### Depression, anxiety, and stress

To measure these variables, the Depression Anxiety and Stress Scale (DASS-21), originally developed by Lovibond and Lovibond [26], translated into German language by Nilges and Essau [27], was used. This questionnaire consists of 21 items with a four-level Likert scale. Seven items each out of the 21 total items measure the participants’ depression, anxiety and stress perception. The advantage of the DASS-21 is that it can be used to assess almost all age groups. Recent research has shown that the results of DASS-21 correlate significantly with the results of other measures that also measure mixed variables such as anxiety and depression. Overall, the DASS-21 is also defined by its validity and reliability [26]. Cronbach’s α was in this sample 0.71 for depression, 0.79 for anxiety, 0.86 for stress, and 0.91 for the total value.

#### Resilience

In this study, the subject's resilience was measured with the resilience scale (RS-11), developed by Schumacher et al. [28]. It assesses factors of resilience such as optimism, emotional stability, joy, energy and openness for new things in one scale [29]. The RS-11 has established itself as a sensible alternative to the long form RS-25. In one study it was found that the differences between the results of the RS-11 and the RS-25 are not significant (difference in mean values = − 0.080; median = − 0.042; effect strength = 0.11). Thus, the use of the RS-11 in research could be recommended as a valid measuring instrument [30]. Furthermore, the internal consistency of the one scale questionnaire was excellent in the present study with a Cronbach’s α of 0.90.

#### Experiences

Initially, the participants were asked to briefly outline their three most significant experiences with teachers in their childhood and/or adolescence. For this purpose, the authors used a self-designed questionnaire. The participants were first informed that it is irrelevant whether they describe a positive or a negative experience. Afterwards, the participants were asked to report on this experience with a teacher in a few sentences. Second, the individual experiences were evaluated in the questionnaire by the participants answering several questions on a five-level Likert scale. They rated (a) the valence of the experience, (b) how motivating it was for them, and (c) how it affected their self-confidence when it happened. Each of the three aspects was evaluated with two items; one of them was reversely coded. For example, from the two questions; how positive was the experience and how negative was it? One total valence score was calculated (after recoding the negative item). The valence score was used to represent the affective quality and the emotional value of an experience for the participants, either on a positive or a negative way. Any questions should be skipped if the respondent can only think of one or two experiences. After every reported experience, the participants were asked to state their age at the time of the experience.

#### Sociodemographic variables

Finally, we collected socio-demographic data such as age and gender, school leaving certificates and professional qualifications with a self-designed questionnaire.

### Procedure

For the data collection, we used an online questionnaire that provided a short introduction and informed the participants about the study. First, we measured participants’ current wellbeing including measures of life satisfaction, stress, anxiety, depression, self-esteem and resilience. Subsequently, the three most significant experiences with teachers in childhood and/or adolescence were reported and evaluated, as described above.

In order to make meaningful use of the participants’ experiences, it was necessary to divide them into different categories to ensure that the individual categories could be compared with each other and to relate the individual types of experiences to the collected variables. In this qualitative evaluation, a summarizing content analysis according to Mayring [31] was used. In the following section, the procedure of this method is described.

### Statistical analysis

#### Qualitative content analysis

To divide the experiences into categories, a passage reading was first performed. On the first day of the content analysis each individual’s experience was read and assigned to a keyword. For example, if a subject reported particularly supportive behaviour on the part of his or her teacher, the researcher noted the keyword “support” for the experience. The keywords were collected in tabular form. The table had two columns, one for positive and the other for negative keywords, respectively. At the same time, we gave every experience a number, so we could note the number and the keyword of the experience in a second table. This way, we could ensure that we could still retrace how each experience was coded later. After coding every experience, the table with the positive and negative keywords was reviewed. While reading through the initial list of positive and negative keywords, it was noticeable that there were some experiences which no keywords were assigned to because they were either too short or consisted of only one word or a random sequence of letters. These records were therefore rated as unusable and invalid. In the next step, we also applied the changes to the table with the numbers of the experiences and their coding.

The next day, the second reading took place. There was a deliberate pause between reading pass one and reading pass two; this ensures that the descriptions already written down were not quite up to date in the researcher’s memories. The second reading and coding session was the same as the first one, and thereafter the first and the second lists were compared. We now checked whether there were differences between the coding of day one and two. If experiences were coded differently, the keywords were checked to see if they described similar behaviors on the part of the teacher or if they implied the same behavior. Significant differences were discussed and reflected on together and a consensus was reached. The next step in the qualitative part of the data evaluation was to combine the collected codes into supercategories. For this purpose, it had to be considered which keywords are similar or could be condensed and attributed to having similar behaviour. In this last step, it was possible to assign all experiences to different categories. These seven categories of experiences we find in our data are individual promotion and support, empathic behaviour, authenticity, unjust and degrading behaviour, physical violence, psychological violence, and sexual assault.

#### Quantitative analyses

SPSS 26.0 was used for all quantitative analyses. As a first step, we calculated the frequencies of each category of experiences and realized a first overview of the data. In order to determine the age structure of the reported experiences, the threshold values had to be calculated, then could the mean age of the participants be determined from all cases. In addition, the percentage shares, the cumulative percentages and the standard deviations were also determined.

The next step was to evaluate the positive and negative valence of the experiences. For this purpose, the questions were used to assess whether the experience was positive or negative for the participant. Furthermore, we evaluated the motivational- and the self-confidence enhancement of the experiences in the questionnaire and calculated the valences.

Subsequently, the data collected on life satisfaction, self-esteem, stress, depression, anxiety, and resilience were evaluated. These results are then compared against the evaluation of the experiences. However, to obtain results regarding the seven categories, further calculations had to be made. First, it was determined again for all three experiences, whether they were positive or negative. However, this was done by assigning them to specific categories. Categories one to three are positive, whereas categories four to seven are negative. The results of these categories were then correlated once more with the variables of life satisfaction, self-esteem, stress, depression, anxiety, and resilience.

In order to evaluate the contribution of the three characteristics of experiences (valence, motivating power, and increasing self-confidence) to well-being and resilience, regression analyses (backword method) were calculated, with the mean values (over three experiences) of valence, motivating power, and increasing self-confidence as predictors, and life satisfaction, self-esteem, total DASS-21, and resilience.

Another important step in the data evaluation was the analysis of variance. The current study was particularly interested in finding out whether the seven categories differ with regard to the variables of life satisfaction, self-esteem, stress, depression, anxiety, and resilience. These differences were illuminated using the analysis of variance and followed by a post-hoc test and testing for inter-subject effects.

## Results

### Characteristics of the sample

A total of n = 157 valid cases were acquired during the data processing. Of the 157 participants, 120 participants (76.43%) are female. Mainly young adults participated in the study (M = 28.17 years; SD = 10.68). The youngest and oldest participants are 19 and 71 years old, respectively.

At the time of the first reported experience, the participants were between five and twenty-four years old (M = 12.72 years; SD = 4.08). The second experience was named by 92.99% of the participants. Their age at the time of the second experience ranged from six to twenty years old (M = 14.21 years; SD = 3.08). The third experience was still described by 75.79% of all participants. At the time of the third experience, the participants were between five and twenty-four years old (M = 15.06 years; SD = 3.24). In all experiences, the average age of the participants was 13.94 years. The mean values show that participants were mostly in their adolescence at the time of their significant experiences.

With regard to the highest school-leaving certificate of the participants, n = 122 (77.71%) indicated that they have passed the Abitur—the German high-school leaving examination. A total of 119 (75.80%) participants were studying for a Bachelor’s or Master’s degree at a University.

### Categories of experiences with teachers

**Fig. 1:**
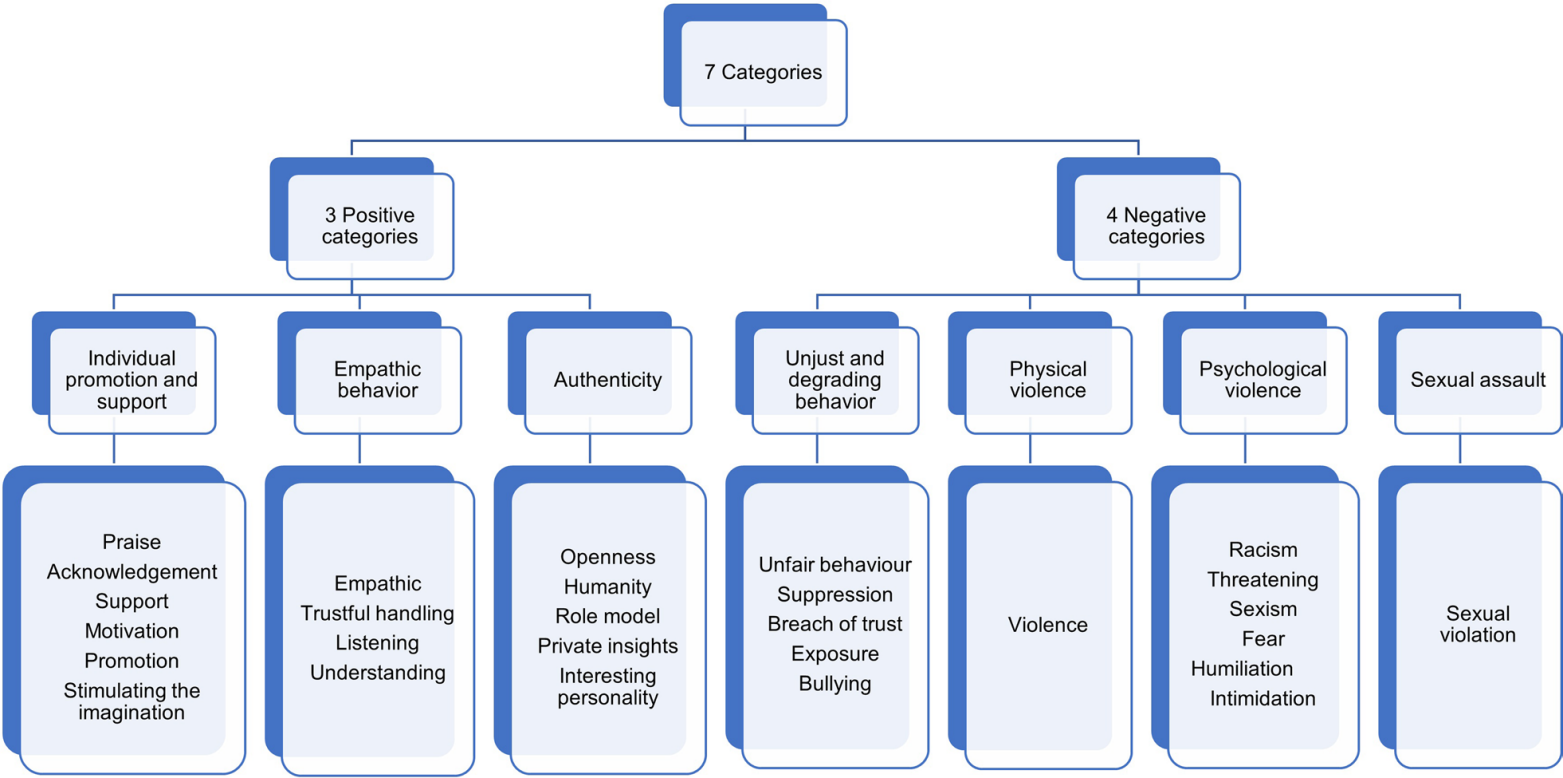
Categories of experiences with teachers

Seven categories resulted from the experiences with teachers in the previously created encodings (see Fig. 1). These are divided into positive (3) and negative (4) categories. The positive categories are individual promotion and support, empathic behaviour, and authenticity. The negative categories are designated as unjust and degrading behaviour, physical violence, psychological violence, and sexual assault.

#### Qualitative results on the categories

##### Individual promotion and support

The category “individual promotion and support” includes experiences that involve special encouragement or support from the teacher. This support goes beyond the minimum requirements of normal school life. In addition, experiences collected here also deal with recognition or praise from the teacher. Finally, in this category, descriptions of special motivation or the stimulation of the student's imagination were condensed. A typical example of an experience from this category could be: “My teacher helped me after school to learn for a class test” or “I never knew exactly what I wanted to do professionally until Mr. X inspired me to become a chemist”.

##### Empathic behaviour

The next category is called “empathic behaviour” and deals with a teacher’s behaviour towards a student that is characterized by trust, understanding, and empathy. The following example could be a typical description from this category: “I told Ms. X about my psychological problems. She listened to me and showed me understanding. At my request, she told no one about these problems.” Another participant described a situation with a math teacher when he/she was 15 years old: “We were supposed to write a math exam, however, the day before, my grandma passed away, and I was feeling very bad. My math teacher noticed this and asked me to step outside and tell him what was going on. I explained the situation and he took me in his arms and tried to build me up a little. After he talked to the other class maids, I was allowed to rewrite the exam”.

##### Authenticity

Another positive category is labelled “Authenticity”. Here, all experiences were assembled, which related to a teacher who showed a high openness towards his/her students. Furthermore, experiences that are listed here also describe a teacher with a particularly interesting personality. Also, descriptions are collected here that outline humanity that goes beyond the norm. Teachers who were labelled as role models for a student or gave some private insight into his/her life are also included in this category. An example in this category from the collected data would be: “I was at a new school and the teacher showed interest in my person. HE (sic) signalled this not only through questions but also non-verbally. In the conversation, he was funny and authentic”.

##### Unjust and degrading behaviour

The first negative category is “unjust and degrading behaviour”. This category summarizes all experiences that entail unfair and degrading memories. Here, memories are listed that describe a type of harassment or bullying. Furthermore, all experiences that showed a breach of trust by the teacher, as well as if the teacher exposed the student or suppressed him/her using his position of authority are placed in this category. An example of the experience in this category is a 16 year-old student’s description of his/her history teacher: “I was always rather the quiet type, and this teacher took you by the hand with the most difficult questions. Often, she also made mean/aggressive remarks. As a result, I seldom attend her class and wanted to participate even less”.

##### Physical violence

Another category that was created is called “physical violence”. In this category, all experiences were collected where a student had experienced physical violence from his/her teacher. The memory of a then ten-year-old child from her primary school days aptly illustrates this category: “The director of the elementary school physically punished me for something I did not do.” Another participant described a memory from primary school when he/she was eight years old: “A primary school teacher used to hit you on the fingers with a curtain rod if you talked to your classmate during a lesson”.

##### Psychological violence

Since the category “unjust and degrading behaviour” did not sufficiently address the severity of some effects, a category for aggravated memories had to be created, called “psychological violence”. This category is distinguished from the category just mentioned by the special quality of the experiences. Therefore, here, memories are collected that tell of intimidation, humiliation, threats, racism, sexism, and teacher’s fomenting of fear in the pupil is listed here. An example for this category is provided by the following description: “The teacher threatened students with a knife and made more racist remarks. I complained and the teacher was suspended. The students were grateful to me”.

##### Sexual assaults

The last category describes sexual assaults by the teacher. Here, the descriptions of actual sexual contact were collected, and those insinuating sexual harassment or the obvious exploitation of the teacher's position to get closer to young people sexually. This category also contains some descriptions that are criminally relevant. For example, one 16 year-old student remembers the following experience: “My sports teacher in the high school used to ask me to show him my dekoleteé (sic) at the beginning of each sports lesson. He always said that he would have to check if I was still wearing jewellery. Even with new exercises I always had to show my classmates—of course under his instruction and “help”—how the new exercise works. All students noticed it; nobody said anything. One girl even told me that I was “exactly his type”. It didn't matter how high-necked my shirts were, it didn't stop. When the swimming lessons were due, I refused to go swimming with this teacher and changed to another course. There I was rated 1 instead of 3^1^”.

##### Invalid cases

Experiences that could not be assigned were classified as invalid. For example, it was frequently the case (n = 80) that experiences were described so briefly that it was not recognizable to which category they could be assigned. An example of this is: “I had to be in detention all the time”. At first glance, one could claim that this was a case of *unjust and degrading behaviour*. However, we do not know from this brief statement whether the detention was perhaps justified and the student had misbehaved. Therefore, this experience cannot be objectively sorted into a category.

#### Quantitative results on the categories

The frequencies of the experiences are presented in Table 1.

**Table 1:** Frequencies of experiences within seven categories

Categories	Exp. 1 (%)	Exp. 2 (%)	Exp. 3 (%)	Total (%)
Individual promotion and support	40 (25.4)	36 (24.7)	44 (36.6)	120 (28.37)
Empathic behavior	12 (7.6)	14 (9.6)	12 (10.0)	38 (8.98)
Authenticity	11 (7.0)	14 (9.6)	2 (1.7)	27 (6.38)
Unjust and degrading behavior	32 (20.4)	40 (27.4)	34 (28.3)	106 (25.06)
Physical violence	10 (6.4)	6 (4.1)	2 (1.7)	18 (4.26)
Psychological violence	15 (9.6)	10 (6.8)	5 (4.2)	30 (7.09)
Sexual assault	2 (1.3)	1 (0.7)	1 (0.8)	4 (0.9)
Invalid	35 (22.3)	25 (17.1)	20 (16.7)	80 (18.91)
Total	157 (100)	146 (100)	120 (100)	423 (100)

### Correlation and regression analyses

#### Correlations with the valence of experiences

The correlations of the valence of the experiences with well-being variables are presented in Table 2. The (positive) valence of the most important experience with teachers (experience 1) is significantly correlated with life satisfaction (r = 0.16, *p* < 0.05) and self-esteem of the participants (r = 0.27, *p* < 0.01). The more positive the participants rated their past experiences in total, the higher their current life satisfaction and self-esteem. The same effect was found between the valence of experience 1 and resilience of the participants (r = 0.21, *p* < 0.05). Also, there is a negative correlation between the valence of experience 1 and depression (r = − 0.20, *p* < 0.05).

With regard to the valence of experience 2, the correlations with self-esteem (r = 0.12) and resilience (r = 0.08) are positive, but lower and not significant (see. Table 1). However, the correlations with anxiety (r = − 0.24, *p* < 0.01) and stress (r = − 0.21, *p* < 0.01) are much higher compared with experience 1. The data indicate that the more negative the second experience, the higher the participants’ current level of fear and stress.

Finally, the mean valence of experiences 1–3 significantly correlate with self-esteem (r = 0.18 *p* < 0.05) and resilience (r = 0.17, *p* < 0.05).

#### Correlations with the motivating power of experiences

The motivating power of experience 1 is correlated with the current self-esteem of the participants (r = 0.27, *p* < 0.01). Also, the motivational effect of experience 2 was highly and significantly correlated with anxiety (r = − 0.26, *p* < 0.01), stress (r = − 0.23, *p* < 0.01), and the DASS-21 Total (r = − 0.23, *p* < 0.01). Furthermore, the resilience of experience 1 (r = 0.19, *p* < 0.05) and the mean resilience (r = 0.20, *p* < 0.05) of participants correlate with the motivational aspect of the teacher-student interaction.

#### Correlations with the self-confidence increased by experiences

Regarding the self-confidence, we found that it is correlated with self-esteem in experience 1 (r = 0.29, *p* < 0.01), experience 2 (r = 0.20, *p* < 0.05) and mean self-esteem (r = 0.24, *p* < 0.01). Once again, the negative correlation with anxiety (r = − 0.27, *p* < 0.01), stress (r = − 0.18, *p* < 0.05) and DASS 21-Total (r = − 0.21, *p* < 0.01) was highly significant. In addition, we were able to find an association between self-confidence enhancing experiences and mean resilience (r = 0.22, *p* < 0.01).

**Table 2:** Correlations of characteristics of the experiences with well-being and resilience

	Valence of the experiences				Motivating power of the experience				Increasing self-confidence			
	Exp. 1	Exp. 2	Exp. 3	Mean	Exp. 1	Exp. 2	Exp. 3	Mean	Exp. 1	Exp. 2	Exp. 3	Mean
Life satisfaction (SWLS)	.16*	.08	− .03	.09	.11	.05	− .02	.04	.15	.04	.00	.06
Self-esteem (SES)	.27**	.12	− .03	.18*	.27**	.16	.05	.21**	.29**	.20*	.06	.24**
Depression (DASS-21)	− .20*	− .12	.06	− .08	− .09	− .13	.03	− .03	− .10	− .14	.05	− .03
Anxiety (DASS-21)	− .11	− .24**	.06	− .12	.01	− .26**	− .07	− .10	.02	− .27**	− .04	− .08
Stress (DASS-21)	− .12	− .21*	− .01	− .14	− .01	− .23**	− .01	− .09	− .02	− .18*	.08	− .04
Psychopathology (DASS-21 Total)	− .15	− .21*	− .04	− .13	− .03	− .23**	− .02	− .08	− .04	− .21**	.04	− .06
Resilience (RS-11)	.20*	.08	− .04	.17*	.19*	.14	.03	.20*	.18*	.16	.08	.22**

*p < .05; **p < .01

#### Regression analyses

In four regression analyses, the explorative question was investigated, which characteristic of the experiences (valence, motivating power, and increasing self-confidence) most strongly predicts well-being and resilience. The results are presented in Table 3. While Increasing self-confidence through the experience remains the only significant predictor of self-esteem and resilience, there is no significant predictor of life satisfaction and psychopathology.

**Table 3:** Regression analyses of characteristics of the experiences as predictors of well-being variables and resilience

Characteristics of the experiences	Life satisfaction (SWLS)			Self-esteem (SES)			Psychopathology (DASS-21 Total)			Resilience (RS-11)		
	B	SE B	β	B	SE B	β	B	SE B	β	B	SE B	β
Mean Valence	.14	.13	.09	− .04	.25	− .02	− .43	.27	− .13	− .05	.40	− .02
Mean Motivating power	− .20	.24	− .12	− .02	.41	− .01	− .17	.73	− .05	.12	.66	.04
Mean Increasing self-confidence	.15	.33	.18	.51	.17	.24**	.48	.50	.13	.76	.27	.22**

**p < .01

### Comparison of the categories of experiences with teachers

The qualitative analysis resulted in three positive and four negative categories of experiences with teachers. In a series of analyses of variance, it was investigated whether these seven categories differed regarding the current satisfaction with life, self-esteem, psychopathology (depression, anxiety, and stress), and resilience. Table 4 presents the means, standard deviations, and results of the ANOVAs including main effect and post-hoc comparisons.

While the seven categories differ significantly regarding life satisfaction (F = 2.6, *p* < 0.05) and self-esteem (F = 4.1, *p* < 0.001), this is not the case for psychopathology (DASS-21 total score; F = 0.48, *p* = 0.82) and resilience (F = 1.6, *p* = 0.14). The post-hoc tests demonstrated that life satisfaction and self-esteem are lowest in two negative categories: “Unjust and degrading behaviour” and “Sexual assault”.

**Table 4:** Means, Standard Deviations, and ANOVA results between the categories of experience 1 regarding life satisfaction, self-esteem, psychopathology, and resilience

	Categories of experience 1									
	1. IPS	2. Emp	3. Auth	4. UDB	5. PHV	6. PSV	7. SA	F	*p*	Sign. diff
Life satisfaction (SWLS)	27.9 (4.3)	29.8 (4.1)	30.0 (1.6)	25.8 (6.0)	28.7 (4.7)	29.1 (2.0)	22.0 (4.2)	2.6	.02*	2 versus 4*, 2 versus 7*, 3 versus 4*, 3 versus 7*, 4 versus 6*, 6 versus 7*
Self-esteem (SES)	23.4 (5.8)	26.4 (2.5)	25.7 (2.2)	20.2 (6.9)	23.5 (5.0)	22.1 (6.1)	10.5 (6.4)	4.1	.001***	1 versus 4*, 1 versus 7**, 2 versus 4**, 2 versus 7***, 3 versus 4**, 3 versus 7**, 4 versus 7*, 5 versus 7**, 6 versus 7**
DASS-21 Total	14.8 (10.5)	13.9 (10.3)	15.1 (9.1)	16.4 (10.4)	14.1 (10.1)	16.5 (14.3)	26.0 (2.8)	.48	.82	–
Resilience (RS-11)	61.9 (11.1)	65.4 (6.8)	64.6 (5.6)	58.8 (9.7)	57.8 (10.0)	64.6 (6.9)	56.0 (4.2)	1.6	.14	2 versus 4 *

*p < .05; **p < .01; ***p < .001, 1. *IPS* Individual promotion and support; 2. *Emp.* Empathic behaviour; 3. *Auth.* Authenticity; 4. *UDB* Unjust and degrading behaviour; 5. *PHV* Physical violence; 6. *PSV* Psychological violence; 7. *SA* Sexual assault

## Discussion

The aim of this study was to examine whether significant experiences with teachers in childhood and adolescence are correlated with well-being in adulthood. The study was able to show that early experiences with teachers are still relevant in adulthood. These experiences are associated with the level of anxiety and stress, resilience, self-esteem, and life satisfaction.

The significance of the results of this study is that in contrast to other studies [1–6, 8–10, 17–19, 21, 32–35] a long-term effect of the experiences could be measured, although this study has only a cross-sectional design. These results have a conceivably high range. It has not yet been known that individual experiences with teachers have such a lasting impact on life or people’s psychological disposition. Since we performed a cross-sectional design, it would be interesting to find out whether a long-term study would lead to similar results. In 2003, Reddy et al. [35] were able to show in a study that a positive teacher-student relationship was related to less depression among students. In the present study, the effect of positive teacher-student interaction could still be demonstrated years later, even if the students are now adults.

Diers [32] showed in 2015 that a supportive and trusting relationship with the teacher is related to higher resilience in students. This study underscores those findings. We found that students with a supportive and empathic relationship with his or her teacher have a higher resilience in adulthood than participants who had an unjust and degrading relationship with a teacher.

Regarding our qualitative analysis we could find a very diverse collection of descriptions and groups of experiences. We were able to find many different positive behaviors from teachers towards their former students, but also plenty of negative ones. We were positively struck by the fact that more than 28% of the experiences were described as promoting, motivational, praising and supporting. On the other hand, 25% of the experiences described were characterized by unjust or degrading behavior, a breach of trust or even bullying. Unfortunately, we found that more than 12% of the experiences clearly described physical or psychological violence and even sexual assault. This shows how differently people experience their school years and what kind of experiences they have along the way.

After evaluating our data collection, we found that more than 70% of the participants were woman. This is a major issue of our data collection and future research should ensure to have a more heterogeneous sample. We think that this bias is due to the fact that we had a high response rate from the mailing lists of the psychological and educational science faculty of the university, where the majority of students is female. This fact may influence the data and our results, because we do not know yet how much the experiences of male and female students differ during childhood and adolescence.

In the future, attention should be drawn to these connections during teacher training so that an awareness of the consequences of individual actions, reactions, and statements can develop in young teachers. This awareness could contribute towards a future outcome in which fewer significant experiences are negative. It has been shown that negative experiences have a particularly strong influence on self-esteem, even if the student–teacher interaction took place a long time ago. The importance of this insight is obvious and, therefore, teachers who are already active should be informed about it so that they can be more reflective when educating their students and in their future professional life. The take home message of our findings for teachers and future teachers is that it should be of high value to be empathetic and supporting towards their pupils. Teachers can achieve this attitude by being interested in student issues and motives, by motivating their students to achieve their goals and by praising them, whenever possible. This leads to an appreciative attitude towards students. This also underscores how responsible and important the teaching profession is.

However, the results of this study also raise further questions. From a clinical perspective it would be worthwhile to examine if psychotherapy is a helpful way to deal with negative experiences with teachers in the past and if it improves factors like self-esteem, resilience, stress, and life satisfaction. Furthermore, future research should consider the influence of experiences with teachers on the accomplishment of developmental tasks. Also, it remains unanswered, if the negative effects of adverse experiences with teachers can be compensated by positive experiences with teachers, peers or parents. Moreover, it would be well worth investigating if the lack of self-esteem and life satisfaction as an adult is associated with a lower socioeconomical status. From the perspective of health sciences these findings raise the question, if negative experiences with teachers might be a predictor of psychosomatic issues as an adult as well. It would be interesting to understand how different experiences interact to influence well-being. It could be considered, for example, whether several negative experiences add up and thus have an even stronger influence on well-being.

### Limitations

Future research building on these results should consider the following issues. First, the sampling of this study was not ideal because a very high proportion of the participants went on to study at the tertiary level, often even pursuing the same course of study at the same university. It would, therefore, be desirable to achieve a broader sample in which there are significantly more participants who did not pursue higher education after high school. Second, the gender distribution should also be more balanced in future studies. Since 76.43% of the participants were woman, we have a selection bias within the cohort. Third, it seems reasonable that in future studies, more researchers should make the categorizations. In this way, the individual experiences could be assigned to the categories even more precisely. Forth, to enhance the impact of the results of this study, a longitudinal study instead of a cross-sectional study should be performed. This would allow the stability of assessing the results. Finally, it seems reasonable to us that people with a high sense of well-being are more likely to remember positive experiences at school than people with a low sense of well-being. This might be a major bias of the data.

## Conclusion

All teachers and teacher training students should be aware of how massive and lasting their influence on their students can be. This study also underlines the importance of teachers not only being professionally qualified for their profession. It is precisely the importance of the pedagogical aspects of teachers’ training that are highlighted by the results of the current article. Teachers should be trained much more intensively in the pedagogical, psychological, and communicative areas. By regularly reflecting on their own behaviour, emotions and thoughts related to students and teaching, teachers can adopt a more supportive attitude which is conducive to and helpful for the development of young people.

The results of this study call for a reflective, fair, authentic, and empathetic approach to students. In this way, a life path can be opened for them that is characterized by a healthy attitude towards themselves and professional success.

## Data Availability

The data that support the findings of this study are available from the corresponding author upon reasonable request.

## Footnotes

1 In the German school-system, 1 is the best and 6 the worst grade.
